# Factors influencing lengths of stay in the intensive care unit for surviving trauma patients: a retrospective analysis of 30,157 cases

**DOI:** 10.1186/cc13976

**Published:** 2014-07-07

**Authors:** Andreas B Böhmer, Katja S Just, Rolf Lefering, Thomas Paffrath, Bertil Bouillon, Robin Joppich, Frank Wappler, Mark U Gerbershagen

**Affiliations:** 1 Department of Anesthesiology and Intensive Care, Hospital Cologne-Merheim, Cologne Medical Center, Witten/Herdecke University, Ostmerheimer Straße 200, Cologne 51109, Germany; 2 Institute for Research in Operative Medicine (IFOM), Witten/Herdecke University, Ostmerheimer Straße 200, Cologne 51109, Germany; 3 Department of Traumatology and Orthopedic Surgery, Hospital Cologne-Merheim, Cologne Medical Center, Witten/Herdecke University, Ostmerheimer Straße 200, Cologne 51109, Germany

## Abstract

**Introduction:**

There are many potential influencing factors that affect the duration of intensive care treatment for patients who have survived multiple trauma. Yet the respective factors’ relevance to ICU length of stay (LOS) has been rarely studied. Thus, the aim of the present study was to investigate to what extent specific factors influence ICU LOS in surviving trauma patients.

**Methods:**

We retrospectively analyzed a dataset of 30,157 surviving trauma patients from the TraumaRegister DGU® who were older than six years of age and received subsequent intensive care treatment for more than one day, from 2002 to 2011. Univariate analysis and multiple linear regression analysis were used to examine 25 categorical pre- and post-trauma parameters.

**Results:**

Univariate analysis confirmed the impact of all analyzed factors. In subsequent multiple linear regression analyses, coefficients ranged from -1.3 to +8.2 days. The factors that influenced the prolongation of ICU LOS most were renal failure (+8.1 days), sepsis (+7.8 days) and respiratory failure (+4.9 days). Patients spent one additional day in the ICU for every 5 additional points on the Injury Severity Score (regression coefficient +0.2 per point). Furthermore, massive transfusion (+3.3 days), invasive ventilation (+3.1 days), and an initial Glasgow Coma Scale score ≤8 (+3.0 days) had a significant impact on ICU LOS. The coefficient of determination for the model was 44% (R^2^).

**Conclusions:**

Treatment regimens, as well as secondary effects and complications of trauma and intensive care treatment, prolong ICU LOS more than the mechanism of trauma or pre-trauma patient conditions. Successful prevention of complicated courses of illness, such as sepsis and renal and respiratory failure, could significantly abbreviate the ICU stay in trauma patients. Therefore, the staff’s attention should be focused on preventive strategies.

## Introduction

Traumatic injuries account for approximately 10% of mortality worldwide [1] and the in-hospital mortality rate of trauma patients in Europe ranges between 15% and 17% [2]. Interventions, such as airway management, blood transfusions and primary surgical care, may be life saving for a trauma patient and can reduce mortality in this patient population [3]. Taking this into account, much focus has been placed on the initial management of the trauma patient. However, even after the initial care in the resuscitation bay or operating theater, patients with severe trauma have a great need for close monitoring and treatment as they are severely injured due to the trauma. Afterwards, those patients are at risk of secondary disorders because of the ongoing pathophysiological reactions that occur after trauma. These necessitate care in the intensive care unit (ICU) to continue resuscitation and manage early post-resuscitation complications [4]. The length of time that intensive care treatment is necessary for those patients who survive multiple trauma might depend on several factors. Conceivable influencing factors are the patient’s pre-trauma status, such as past medical history, present illnesses, age and gender [5], and the trauma itself, circumstances of the accident, injury severity, pattern of injuries and so on [6]. For example, pre-injury polypharmacy was recently shown to be a predictor of trauma outcome and was related to an extended ICU length of stay (LOS) [7]. However, pre-trauma status, as well as trauma itself, cannot be influenced by the medical staff’s efforts.

Furthermore, treatment regimens, conservative as well as surgical, transfusion strategies and so on [8] might be factors that influence LOS. At the very least, trauma, as well as intensive care treatment, can influence the occurrence of secondary effects, such as sepsis or multi-organ failure [9], leading to an increased duration of ICU stay. Moreover, patients with major trauma are at highest risk for venous thromboembolism resulting in an increased LOS [10]. The latter, treatment regimens and secondary effects, can be influenced by the ICU staff.

Mean LOS in the ICU after severe trauma was found to be approximately 8 days in Germany (survivors and non-survivors included) [11]. Clinicians may feel that there are several factors that can prolong ICU LOS in multiple-trauma patients. However, the extent to which the respective factors influence ICU LOS in patients who survive multiple trauma has rarely been studied and the impact on clinical practice has yet to be determined. Therefore, the aim of the present study was to provide detailed data, defined by a specified number of days, on factors that prolong or reduce ICU LOS in a large cohort of surviving trauma patients.

## Methods

### Data collection

In the present study, we analyzed data from the German TraumaRegister DGU® (Deutsche Gesellschaft für Unfallchirurgie, German Trauma Society) database. The TraumaRegister DGU® is a prospective multi-center database with standardized and anonymous documentation of severely injured patients who have experienced trauma and thus require admission to an intensive care unit. Data were collected from the point of accident with subsequent stay in the ICU or intermediate care unit to clinical discharge [2]. Preclinical deaths, burns, poisonings and femur neck fractures in elderly people were not included. This registry comprises detailed information on demographics, patients’ pre-existing conditions, trauma mechanism, treatment and clinical course, and clinical and laboratory data, as well as a variety of standardized scoring systems on injury severity, such as the Glasgow Coma Scale (GCS), the Injury Severity Score (ISS) and the Abbreviated Injury Score (AIS). A total of 81,622 patients from 407 participating hospitals from 2002 to 2011 were included.

### Ethical bodies

The TraumaRegister DGU® is a voluntary registry, and participation is free of charge. As a compulsory tool for quality assessment, no informed consent is necessary for data collection. However, participating hospitals agree to scientific evaluation of contributed data that has been de-identified. Prior to dataset analysis, scientists have to apply to use data in written form, which explains the key question and scientific background of the project. After approval by the institutional review board, the study will be registered and results as well as its publication will be reviewed internally and recorded [12].

The infrastructure for documentation, data management and data analysis is provided by the Academy for Trauma Surgery (AUC - Akademie der Unfallchirurgie GmbH), a company affiliated to the German Trauma Society. The scientific leadership is provided by the Committee on Emergency Medicine, Intensive Care and Trauma Management (Sektion NIS) of the German Trauma Society. The participating hospitals submit their data anonymously into a central database via a web-based application. Participation in TraumaRegister-DGU® and analysis of data are approved by the participants’ institutional ethical review boards. Institutional ethical review board agreement documents were not administered by TraumaRegister DGU®.

Scientific data analysis is approved according to a peer-review procedure established by Sektion NIS. The TraumaRegister DGU® is approved by the review board of the German Trauma Society and is in compliance with the institutional requirements of its members. The present investigation has been approved and registered under DGU 2012–049 by the institutional review board of the DGU.

### Patients

**Figure 1:**
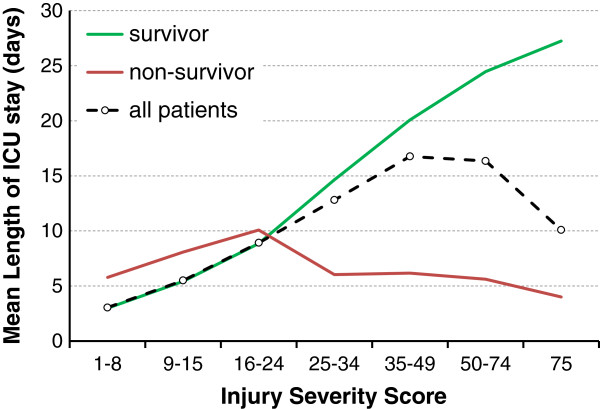
Development of ICU LOS for survivors, non-survivor population and all patients according to injury severity.

The registry was searched for patients who were treated between 2002 and 2011 in Germany who stayed for more than one day in the ICU. To avoid large deviation from the mean LOS, those patients who stayed longer than 90 days in the ICU were excluded (0.2% of the patient total). Furthermore, children under the age of six years and those patients who had been transferred in from another hospital were excluded. Because LOS in the ICU does not have a linear relationship with injury severity (Figure 1), the detailed analysis of factors influencing LOS was limited to survivors of trauma only. Non-survivors show an inverse association with LOS due to the increasing number of early deaths. To consider the whole population (survivor plus non-survivor) would therefore mask severity-dependent factors.

### Parameters

We analyzed 25 parameters that can be divided into four different sections: epidemiological parameters (‘the patient’); parameters directly associated with the trauma incident (‘the accident’); the initial emergency treatment; and parameters that represent complications or sequelae, which can occur hours or even days after trauma.

Gender and age were determined to be the epidemiologic parameters. All analyzed parameters were expressed as categorical variables, except for the ISS, which was expressed as a continuous variable. Age was grouped as a categorical parameter as follows, children (age 6 to 15 years) and ages 16 to 49 years, 50 to 59 years, 60 to 69 years, 70 to 79 years, and 80 years and older. The pre-trauma American Society of Anesthesiologists (ASA) classification was used to document significant pre-existing diseases (ASA score ≥3). To examine the trauma itself, an initial pre-hospital GCS ≤8, the ISS, and the severity of injury of the head, thorax, abdomen and extremities as assessed with an AIS ≥3 were used. High energy trauma (car or motorcycle accident, and fall from a height >3 m) was considered as well.

In the acute care section, we considered conservative versus operative therapy, damage control surgery was conceptualized as the first surgical intervention, multiple operations was defined as five operations or more, need of invasive ventilation, blood transfusion and massive transfusion, which was defined as ≥10 units of packed red blood cells (PRBC). As secondary effects of trauma and intensive care, we assessed the incidence of sepsis, multi-organ failure and, separately, organ failure of the respiratory system, cardiovascular system, central nervous system (CNS), coagulation system, liver, and kidneys. Organ failure was defined according to the Sequential Organ Failure Assessment score [13] where a score ≥3 for at least two days was considered as organ failure (Table 1).

**Table 1:** Criteria of organ failure of single organ systems as used in the study according to the Sequential Organ Failure Assessment

**Organ**	**Definition of organ failure**
Lung	PaO_2_/FiO_2_ < 200 and mechanically ventilated
Heart	dopamine >5 μg/kg/min or any administration of (nor-)/epinephrine
Central nervous system	Glasgow Coma Scale ≤9
Coagulation	platelets <50/nl
Liver	bilirubin ≥6.0 mg/dl
Kidney	creatinine ≥3.5 mg/dl or urine output <500 ml/d

The criteria must be met for at least two days to be considered organ failure.

As mentioned above, we included three parameters describing loss of consciousness and head injury, respectively: An initial GCS ≤8, which describes the GCS at the time of preclinical examination by emergency staff. An AIS score for the head ≥3 was documented as the final diagnosis and defines the severity of tissue damage. Organ failure of the CNS was defined as a GCS ≤8 for at least two consecutive days and was supported by the continuation of CNS impairment.

Because traumatic injury is associated with coagulopathy, which is considered to be multifactorial [8], we summarized relevant pathological findings, coded in the registry [12] (hemoglobin, base excess, platelet count and prothrombin time, Table 2). If at least one factor that was observed on admission met our arbitrarily, predefined criteria, we categorized this patient positive for this complex, which we called ‘hematological disturbance’. Thus, organ failure of the coagulation system, which was used as a screening parameter during the intensive care period, could be extended with hematological disturbances on admission.

**Table 2:** Definition of hematological disturbance (at least one condition measured on admission)

**Factor**	**Finding**
Hemoglobin	≤8 g/dl
Base excess	≤-6 mmol/l
Platelet count	≤80/nl
Prothrombin time	≤55%

Finally, we divided the dataset into two time periods (2002 to 2006 and 2007 to 2011) to detect any development in LOS over time.

### Statistical analysis

Categorical variables were presented as number of cases and percentage, and mean values were given for continuous variables, as well as the number of valid cases. Standard deviation (SD), range or median are given as appropriate. Univariate analysis with formal statistical testing was performed with the chi-square test or Mann-Whitney *U* test. After univariate analysis, a multiple linear regression analysis was conducted with ICU length of stay as the dependent variable. The regression coefficients for each variable could thus be interpreted as number of days in the ICU. Coefficients are presented together with their respective standard error (SE) and level of significance.

To keep our analysis clear, calculation of the interaction of related terms was not performed due to the large number of possibilities of interaction given 25 predictors.

The overall percentage of explained variance of the model was described by the adjusted R^2^ of observed and predicted LOS. We analyzed observed versus predicted LOS for supraregional and regional hospitals. Due to the large number of cases, the detectable difference with 30,000 cases is very small (approximately 0.02 SD), which corresponds approximately to a quarter of a day. Therefore, ‘significance’ should be interpreted with caution.

All analyses were performed with SPSS statistical software package (version 20, IBM Inc., Armonk, NY, USA).

## Results

A total of 33,338 patients from 164 trauma centers met our inclusion and exclusion criteria. Among these there were 3,181 non-survivors with an average LOS of 9.4 (median 5) days. The ICU LOS was linearly associated with injury severity only in survivors, not in all cases (see also Methods section and Figure 1). After exclusion of non-survivors, 30,157 patients were enrolled for further analysis. Their average LOS in the ICU was 11.5 (median 7) days and 26.3% were female and 73.7% were male. The mean age was 43 ± 20 years and the mean ISS was 21.9 ± 11.7. Basic demographic and clinical characteristics are described in Table 3. Injury patterns were as follows (counted for AIS ≥ 3): 42.3% of patients had a head injury, 47.0% had an injury of the thorax, 15.8% had an injury of the abdomen, and 32.6% had an injury of the extremities. Ninety-five percent of the patients had a blunt trauma, and 4.9% had a penetrating trauma.

**Table 3:** Basic demographics and clinical characteristics of patients analyzed

	**Valid cases**^**a**^	**Mean ± SD**	**Median**	**Range**
Age (years)	30,157	43.4 ± 19.9	42	6 to 103
ISS	30,157	21.9 ± 11.7	20	1 to 75
Invasive ventilation (d)	29,945	6.5 ± 10.1	2	0 to 120
ICU LOS (d)	30,157	11.5 ± 12.0	7	2 to 90
Hospital LOS (d)	29,990	27.2 ± 24.0	21	2 to 362

^a^Absolute number of cases with information on specific parameter available in the registry. ICU, intensive care unit; ISS, Injury Severity Score; LOS, length of stay; SD, standard deviation.

In the univariate analysis, each factor was found to be significantly associated with ICU LOS (Table 4). Because the result of the constant of the multiple linear regression analysis was <0.5 (0.4, SE 0.219), we interpreted the data disregarding the constant. Therefore, every regression coefficient in the multiple linear regression analysis (Table 5) can be interpreted as the number of days that a patient has to stay longer or shorter due to a single parameter.

**Table 4:** **Results of univariate analysis**^**a**^

	**Parameter**	**Valid cases**^**b **^**(n)**	**Number of cases**^**c**^	**Percentage of cases (%)**	**ICU LOS ± SD; ME (days)**
Age				
80+	30,157	1,345	4.5	11.2 ± 12.6; 6
70 to 79		2,505	8.3	14.0 ± 14.2; 9
60 to 69		3,076	10.2	13.0 ± 13.2; 8
50 to 59		4,110	13.6	12.1 ± 12.7; 7
16 to 49		18,025	59.8	11.0 ± 11.2; 6
6 to 15		1,097	3.6	8.1 ± 8.5; 4
Gender				
Male	29,961	22,077	73.7	11.6 ± 12.1; 7
Female		7,884	26.3	10.7 ± 11.5; 6
ASA score				
≥3	28,921	6,150	21.3	13.2 ± 13.3; 9
<3		22,771	78.7	11.0 ± 11.5; 6
Treatment year				
2007 to 2011	30,157	20,536	68.1	11.1 ± 11.7; 6
2002 to 2006		9,626	31.9	12.1 ± 12.4; 8
High energy				
Yes	22,050	14,607	66.2	12.8 ± 12.3; 8
No		7,443	33.8	12.2 ± 12.6; 7
AIS head				
≥3	30,157	12,894	42.8	14.5 ± 12.9; 11
<3		17,264	57.2	9.2 ± 10.6; 5
AIS thorax				
≥3	30,157	14,164	47.0	13.9 ± 12.9; 10
<3		15,994	53.0	9.4 ± 10.6; 5
AIS abdomen				
≥3	30,157	4,772	15.8	14.8 ± 14.1; 10
<3		25,386	84.2	10.9 ± 11.4; 6
AIS extremities				
≥3	30,157	9,846	32.6	13.2 ± 13.2; 9
<3		20,312	67.4	10.6 ± 11.2; 6
Initial GCS				
≤8	28,272	6,573	23.2	17.6 ± 13.5; 15
>8		21,699	76.8	9.7 ± 10.8; 5
Hematological disturbance				
Yes	27,566	6,673	24.2	16.0 ± 14.1; 12
No		20,893	75.8	10.2 ± 10.9; 6
Transfusion				
≥10	30,157	1,167	3.9	23.9 ± 16.4; 21
<10		4,754	15.8	16.3 ± 13.4; 13
No		24,241	80.4	9.9 ± 10.7; 5
Invasive ventilation				
Yes	30,157	20,961	69.5	14.5 ± 12.9; 11
No		9,201	30.5	4.6 ± 4.7; 3
Conservative treatment				
Yes	22,772	184	0.8	14.1 ± 13.1; 10
No		22,588	99.2	12.5 ± 12.4; 8
Damage control surgery				
Yes	30,157	9,116	30.2	14.1 ± 13.2; 10
No		21,046	69.8	10.4 ± 11.2; 6
Number of operations				
≥5	22,772	7,058	31.0	17.0 ± 14.5; 13
<5		15,714	69.0	10.6 ± 10.8; 6
Sepsis				
Yes	28,237	2,271	8.0	27.5 ± 15.7; 25
No		25,966	92.0	10.0 ± 10.4; 6
Multi-organ failure				
Yes	27,996	6,007	21.5	22.1 ± 14.6; 19
No		21,990	78.5	8.6 ± 9.3; 5
OF respiratory				
Yes	27,996	5,513	19.7	22.0 ± 14.7; 19
No		22,483	80.3	8.9 ± 9.3; 5
OF cardiovascular				
Yes	27,996	6,236	22.3	20.1 ± 14.4; 17
No		21,760	77.7	9.0 ± 10.0; 5
OF CNS				
Yes	27,996	5,009	17.9	19.9 ± 14.0; 17
No		22,987	82.1	9.7 ± 10.7; 5
OF coagulation				
Yes	27,996	2,083	7.4	21.5 ± 16.4; 18
No		25,913	92.6	10.7 ± 11.2; 6
OF liver				
Yes	27,996	481	1.7	29.2 ± 17.9; 25
No		27,515	98.3	11.2 ± 11.6; 7
OF kidneys				
Yes	27,996	708	2.5	30.6 ± 20.1; 27
No		27,288	97.5	11.0 ± 11.3; 6

^a^Formal statistical testing with Mann-Whitney-*U*-test: all tested parameters had significant influence on ICU LOS (p <0.05). ^b^Number of cases with information on specific parameter available. ^c^Number of cases in specific category. AIS, Abbreviated Injury Score; ASA, American Society of Anesthesiologists; CNS, central nervous system; GCS, Glasgow Coma Score; ICU LOS, mean intensive care unit length of stay in days; ME, median; OF, organ failure; SD, standard deviation.

**Table 5:** Results of linear regression analysis

	**Parameter**	**Regression coefficient (SE)**
Pre-trauma	age 80+	2.2 (0.390)**
	age 70 to 79	3.3 (0.282)**
	age 60 to 69	2.3 (0.242)**
	age 50 to 59	1.5 (0.209)**
	age 16 to 49	reference group
	age 6 to 15	-1.2 (0.398)*
	Gender	n.i.
	ASA	1.1 (0.179)**
	Treatment after the year 2007	-1.3 (0.149)**
Trauma	High energy	n.i.
	AIS head	1.3 (0.176)**
	AIS thorax	n.i.
	AIS abdomen	n.i.
	AIS extremities	-1.0 (0.161)**
	Initial GCS	3.0 (0.190)**
Post-trauma	Hematological disturbance	1.2 (0.166)**
	Transfusion	0.7 (0.190)**
	Massive transfusion	3.3 (0.349)**
	Invasive ventilation	3.1 (0.185)**
	Conservative treatment	2.7 (0.834)*
	Damage control surgery	n.i.
	Multiple operations	2.8 (0.171)**
	Sepsis	7.8 (0.258)**
	Multi-organ failure	n.i.
	OF respiratory	4.9 (0.194)**
	OF cardiovascular	1.5 (0.190)**
	OF CNS	2.1 (0.211)**
	OF coagulation	n.i.
	OF liver	3.9 (0.511)**
	OF kidneys	8.1 (0.440)**

Regression coefficients could be interpreted as days of ICU stay. *P <0.01, **P <0.001. AIS, Abbreviated Injury Score; ASA, American Society of Anesthesiologists; CNS, central nervous system; GCS, Glasgow Coma Score; n.i., not included in linear regression analysis (non-significant coefficient); OF, organ failure; SE, standard error.

Because the ISS showed linearity in the prolongation of LOS (+0.2 days per ISS point), it was examined as a continuous parameter. Using multiple linear regression analysis, the following parameters were found to have significant influence on LOS in the ICU.

Considering the demographic variables, each age group older than 50 years had a prolonged LOS of 1.5 days to 3.3 days, whereas childhood was seen to reduce LOS by 1.2 days. ASA classification demonstrated a mild effect on ICU LOS (+1.1 days).

Among the trauma parameters, GCS ≤8 led to 3.0 additional days in the ICU. AIS for injuries of the head extended LOS in the ICU (+1.3 days), whereas AIS for injuries of the extremities had an abbreviating effect on ICU LOS by 1.0 day.

Even though conservative therapy and treatment with multiple operations represent different strategies, both resulted in a similar impact on LOS (+2.7 days and +2.8 days, respectively). Need for invasive ventilation occurred in 69.5% of the cases (Table 4) and extended ICU stay on average by 3.1 days. Transfusion, massive transfusion and hematological disturbance increased the need for intensive care (+0.7 days, +3.3 days and +1.2 days, respectively). Among secondary effects, respiratory (19.7%; +4.9 days), cardiovascular (22.3%; +1.5 days) and CNS failure (17.9%; +2.1 days) occurred most frequently. However, sepsis and kidney failure had a higher impact on the ICU LOS (+7.8 days and +8.1 days, respectively). Overall, there was a trend of reduced LOS in the ICU in recent years (treated from the year 2007: -1.3 days).

**Figure 2:**
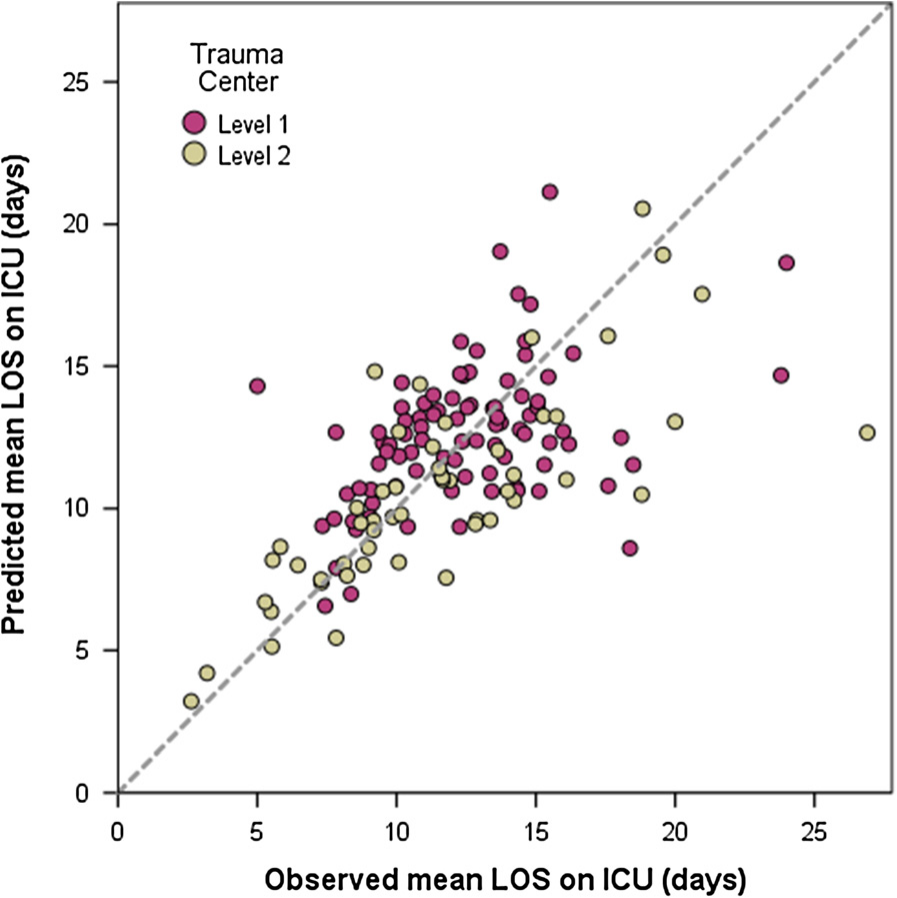
**Predicted ICU LOS versus observed LOS for included trauma centers.** Hospitals contributing ≥5 cases to the dataset were included (148 out of 164). Level 1 = supraregional hospital; Level 2 = regional hospital.

The remaining parameters (gender, age 16 to 49 years, AIS of thorax and abdomen, high energy trauma, damage control surgery, multi-organ failure, and organ failure of coagulation) were not found to independently influence the ICU LOS. The coefficient of determination of this model was R^2^ = 44%, thus nearly half of the observed variance could be explained by the factors analyzed.Predicted versus observed LOS is shown in Figure 2. In the group of supraregional hospitals, the predicted mean LOS matched the observed LOS (predicted 12.6 days, observed 12.5 days). In regional hospitals, the observed mean LOS was 0.9 days longer than the predicted LOS (predicted 10.5 days, observed 11.4 days).

## Discussion

The reduction of ICU LOS should be a significant aim of medical staff because longer ICU stays increase the risk for infection or delirium, are associated with a higher risk of hospital death [14,15], and have immense costs [16]. To our knowledge, this study is the first attempt to provide estimates for additional ICU LOS according to individual conditions or complications in a large cohort of trauma patients.

The regression coefficients calculated from these data can be interpreted as the number of days prolonging or abbreviating ICU LOS, depending on their sign (positive or negative, respectively). For each patient, the coefficients, if applicable, could thus be added to give an estimated length of stay, including a basic number of days, which is the constant term in the model. To describe how well the estimated LOS agrees with the observed LOS, a correlation coefficient R was calculated. The coefficient of determination, which is defined as the squared correlation coefficient (R^2^), was 0.44. This means that 44% of the observed variance could be explained by the analysis, which is a good result. For comparison, an observational study of 11,295 patients that evaluated ICU LOS risk-adjusted models revealed that APACHE IV - a model originally developed for the prediction of both mortality and ICU LOS - had a coefficient of determination of 0.202, whereas the Simplified Acute Physiology Score II’s coefficient of determination was 0.008 [17]. In light of these findings, our analysis provides a good balance between accuracy and data burden.

We included two types of parameters in our analysis. On one hand, there were parameters that cannot be modified by the efforts of intensive care staff, such as patients’ age, trauma mechanisms and related injury severity. On the other hand, we analyzed secondary effects of trauma during intensive care. Interestingly, post-trauma or secondary effects of trauma and intensive care treatment are parameters that prolong ICU stay to the greatest extent. In contrast to patients’ pre-trauma condition or trauma mechanisms, those secondary effects can be influenced by staff behavior. For example, specific ventilator strategies are assumed to prevent acute respiratory distress syndrome (ARDS) [18], there are several strategies reported to prevent central line-associated bloodstream infections [19], and fluid resuscitation using colloids with a risk of increased mortality is an issue of discussion [20-22]. Therefore, by avoiding secondary effects, extended stays in the ICU might be considered preventable. As these secondary effects can also be attributed to preclinical or emergency room treatment, this should be attended to by ICU, as well as preclinical or emergency room staff. Keeping a trauma patient’s ICU stay as short as possible reflects a hospital’s quality of care and attitude toward safety. Taking this into account, further research should focus on those strategies that sufficiently reduce complications, such as sepsis or organ failure, to reduce LOS in trauma patients.

Among pre-trauma and epidemiologic patient parameters, the most prominent parameter to prolong ICU stay was patients’ age. This is in accordance with recent findings on general hospital LOS in the elderly, which is 10 days longer than that of the non-elderly population [5]. The average age of patients with trauma in Europe has increased over the last decade [23] following the demographic trend. Taking this into account, one might assume that some medical efforts in trauma therapy, which may contribute to LOS reduction, may partially be limited by changes in the population characteristics of trauma patients.

While trauma mechanism itself had no effect, injury severity influenced LOS. In our study, every five points on the ISS counted for one additional day in the ICU. This underlines the relevance of ISS as a predictor of outcome after trauma [6].

Three parameters in our analysis dealt with head injury and loss of consciousness, respectively. The initial GCS score showed most relevance for LOS. According to our results, LOS can be extended by more than six days due to head injury (+3 days initial GCS, +2.1 days for organ failure of CNS, +1.3 days AIS head). In trauma patients with a GCS ≤8, the establishment of an airway is recommended [24], which is accompanied by need for mechanical ventilation and therefore can increase the ICU LOS. Furthermore, the need for deep sedation and possible neurological deficits might prolong the ICU LOS in patients with head injury. The results of our analysis confirm the importance of head injury in trauma patients as it relates to ICU LOS.

Treatment regimens were observed to be of relevance for ICU LOS. Because hemorrhage is a dominant risk for traumatized patients [25], transfusion management remains a challenging task. Recently, the importance of early and balanced use of blood products in severely injured patients was emphasized to reduce mortality and total blood loss [26]. Controversially, early transfusion of PRBC increases the risk for ARDS [27], as well as multi-organ dysfunction syndrome and nosocomial infection [28].

Coagulopathy develops due to several factors, such as tissue injury, massive transfusion, and consumption of clotting factors and platelets [29]. Our study revealed that just one single abnormal blood test result on admission, according to our definition, indicates one additional ICU day. Regarding extended ICU LOS, coagulopathy should be treated appropriately [30], and the amount of PRBC should be reduced if possible by, for example, considering early administration of tranexamic acid [31].

Albeit a rare complication within the evaluated population, renal failure increased the ICU LOS to the greatest extent among the second group of influencing factors. Renal failure may be a result of sepsis or multi-organ failure; nevertheless, in this study it was an independent variable for ICU LOS prolongation. Our findings are consistent with data of a general hospital population, in which even a serum creatinine concentration increase of 0.5 mg/dl was found to prolong hospital LOS by 3.5 days [32]. Our data underline the impact on ICU LOS if renal failure occurs after trauma. Patients with severe renal failure often need renal replacement therapy and have increased risk for cardiovascular complications, which might decrease the feasibility of discharge from the ICU and could be an explanation for prolonged ICU stay.

Trauma patients are known to have an increased risk for sepsis, with an incidence between 2% and 8% [33]. Among secondary effects of trauma, sepsis was one of the prominent causes for extending LOS in the ICU. Our data were supported by the results of several studies that show a prolongation of ICU LOS due to bloodstream infection and sepsis [33]. They underline the need for further studies to reduce risk factors for sepsis in patients with trauma. These may include strategies and protocols to change staff members’ safety attitudes to avoid the occurrence of infections [34], as well as approaches to modulate post-traumatic immune depletion [35] or cytokine absorption [36].

Respiratory failure was defined similar to moderate ARDS criteria (Table 1). ARDS increases the median duration of mechanical ventilation [37], which can be correlated with longer ICU stays. In trauma patients, ARDS is associated with longer hospital and ICU LOS, and increased morbidity, as well as costs, whereas mortality is not affected [38]. In blunt trauma, independent risk factors for ARDS were ISS ≥25, pulmonary contusion, age ≥65 years, hypotension on admission and massive transfusion [39]. Early transfusion especially promotes ARDS, while each unit of PRBC increases the risk by 6% [27], which might lead to more conservative transfusion strategies. The prevention of respiratory failure can reduce ICU LOS by 43% (-4.9 days; 6.6 days instead of 11.5 days), which could be supported by lung protective ventilation using low tidal volumes or the use of positive end expiratory pressure ventilation, among other strategies [40].

Our study confirms the prolonging effect of invasive ventilation on ICU stay. The risk for complications, such as pneumonia or airway trauma, increases with length of ventilation [41], which again is correlated with a longer ICU stay [42]. Early tracheotomy can be considered to decrease time of mechanical ventilation and ICU LOS [43] because it alleviates spontaneous ventilation. However, our study did not discriminate between the modes of invasive ventilation or whether intubation or tracheostomy was established.

The comparison between supraregional and regional hospitals revealed a variation in predicted versus observed LOS. According to our data, one might consider differences in organizational structure, transfer management or treatment regimen between these types of hospitals leading to prolonged or shortened LOS. However, our study did not analyze treatment regimen in detail. Further studies are required to explain the deviations between observed LOS and calculated LOS.

The strength of our data is that it provides a quantification of factors’ influence on the ICU LOS in patients with multiple trauma. These results are useful because they can be used for benchmarking as well as quality assurance. Our data give a direction for clinicians to determine to what extent the ICU LOS can be influenced by modification of the frequency of particular complications.The study had several limitations. First of all, typical pitfalls of registry analysis, such as completeness of reporting, different policies in care and so on should be taken into consideration. This study represents a ‘European’ trauma population with a large proportion of blunt trauma incidents. Our results might be influenced by the underlying trauma mechanisms and, therefore, its application to other trauma populations might be limited. Exclusion of non-survivors might be interpreted as a limitation because in-hospital death itself undoubtedly influences ICU LOS. Our motivation for analyzing the survivor population only was driven by the fact that in the total group, LOS was associated with severity in a non-linear fashion. Interventions, such as mass transfusion, that are supposed to prolong the ICU treatment might turn out to shorten it if many early deaths were found in these subgroups. Thus, we decided to focus on the analysis of specific parameters in the subgroup of surviving trauma patients only. In this subgroup, LOS in the ICU increased with severity, as expected (Figure 1). Although ICU LOS is usually not normally distributed, we used multiple linear regression to keep up interpretability of results as number of days. In addition, quality of diagnosing and reporting was not measured.

The factors included in our analysis may interrelate, although a multivariate analysis was performed. For instance, a severely injured patient with a high ISS will be vulnerable to complications, such as the occurrence of sepsis or ARDS, which we have shown to influence the ICU LOS decisively. Furthermore, occurrence of sepsis itself is a risk factor for acute renal failure, which again prolongs LOS. As mentioned before, we decided against analyzing interaction related terms to maintain applicability. However, although the grade of interrelation remains vague, all factors influencing LOS are relevant as independent factors.

Furthermore, although the coefficient of determination is satisfactory, it has to be acknowledged that the true LOS could still be determined by other factors not included or measured in our analysis. Nevertheless, we were able to explain nearly half of the observed variance. Moreover, different aspects of preclinical and emergency room management, such as intubation at the scene or volume management, were not included. A prospective approach that focuses much more on details during the intensive care unit stay would be desirable to detect further relevant factors influencing the ICU LOS.

## Conclusion

Many factors influence ICU LOS in surviving patients with trauma. However, only some of them can be influenced and modified by the intensive care team. To increase trauma patients’ safety and quality of care, ICU staff as well as preclinical and emergency room staff should focus on those factors and develop preventive strategies.

## Key messages

• Treatment regimens, as well as secondary effects and complications of trauma and intensive care treatment, prolong ICU LOS more than the mechanism of trauma or pre-trauma patient conditions.

• The most prominent epidemiological parameter to prolong ICU stay was patients’ age.

• Secondary effects that influenced the prolongation of ICU LOS most were renal failure, sepsis, and respiratory failure.

• Every five points on the ISS counts for one additional day in the ICU.

• Successful prevention of complicated courses of illness could significantly abbreviate the ICU stay in trauma patients.

## Abbreviations

AIS: Abbreviated Injury Score; ARDS: acute respiratory distress syndrome; ASA: American Society of Anesthesiologists; CNS: central nervous system; DGU®: Deutsche Gesellschaft für Unfallchirurgie, German Trauma Society; GCS: Glasgow Coma Scale; ICU: intensive care unit; ISS: Injury Severity Score; LOS: Length of stay; OF: organ failure; PRBC: packed red blood cells; SD: standard deviation; SE: standard error.

## Competing interests

The authors declare that they have no competing interests. This study was realized without any financial support.

## Authors’ contributions

ABB and KSJ designed the study, participated in the statistical analysis and drafted the manuscript. RL performed the statistical analysis, participated in the study design and helped to draft the manuscript. FW helped to design the study and to draft the manuscript. MUG helped to design the study and to draft the manuscript. RJ, TP and BB contributed to the study design and revision of the article. All authors read and approved the final manuscript.
